# New *Ca*. Liberibacter psyllaurous haplotype resurrected from a 49-year-old specimen of *Solanum umbelliferum*: a native host of the psyllid vector

**DOI:** 10.1038/s41598-019-45975-6

**Published:** 2019-07-02

**Authors:** Kerry Elizabeth Mauck, Penglin Sun, Venkata RamaSravani Meduri, Allison K. Hansen

**Affiliations:** Department of Entomology, University of California, Riverside, 900 University Ave, Riverside, CA USA

**Keywords:** Haplotypes, Bacterial genetics

## Abstract

Over the last century, repeated emergence events within the *Candidatus* Liberibacter taxon have produced pathogens with devastating effects. Presently, our knowledge of *Ca*. Liberibacter diversity, host associations, and interactions with vectors is limited due to a focus on studying this taxon within crops. But to understand traits associated with pathogen emergence it is essential to study pathogen diversity in wild vegetation as well. Here, we explore historical native host plant associations and diversity of the cosmopolitan species, *Ca*. L. psyllaurous, also known as *Ca*. L. solanacearum, which is associated with psyllid yellows disease and zebra chip disease, especially in potato. We screened tissue from herbarium samples of three native solanaceous plants collected near potato-growing regions throughout Southern California over the last century. This screening revealed a new haplotype of *Ca*. L. psyllaurous (G), which, based on our sampling, has been present in the U.S. since at least 1970. Phylogenetic analysis of this new haplotype suggests that it may be closely related to a newly emerged North American haplotype (F) associated with zebra chip disease in potatoes. Our results demonstrate the value of herbarium sampling for discovering novel *Ca*. Liberibacter haplotypes not previously associated with disease in crops.

## Introduction

Plant pathogen emergence is a major threat to food security^1^ and occurs through multiple, non-mutually exclusive pathways^2^. For example, a plant pathogen may be a previously unknown or undetected organism, or an organism that was once non-pathogenic to its host but has evolved pathogenic traits over time. Alternatively, the plant pathogen may already be known, but has spread to, and proliferated within, a new geographic area or host population. An outbreak may also represent the re-emergence of a plant pathogen whose incidence had declined significantly in the past but, more recently, increased over a short period. These pathways of disease emergence are complex, and thus require consideration of microbe-plant interactions in both managed and unmanaged systems. With advances in sequencing technologies and development of methods to overcome detection challenges in non-crop hosts^3^, inclusion of wild vegetation in microbial diversity and pathogenicity studies is enabling the discovery and characterization of novel strain types^4^, and expanding our understanding of the processes underlying pathogen emergence^5–8^.

**Figure 1:**
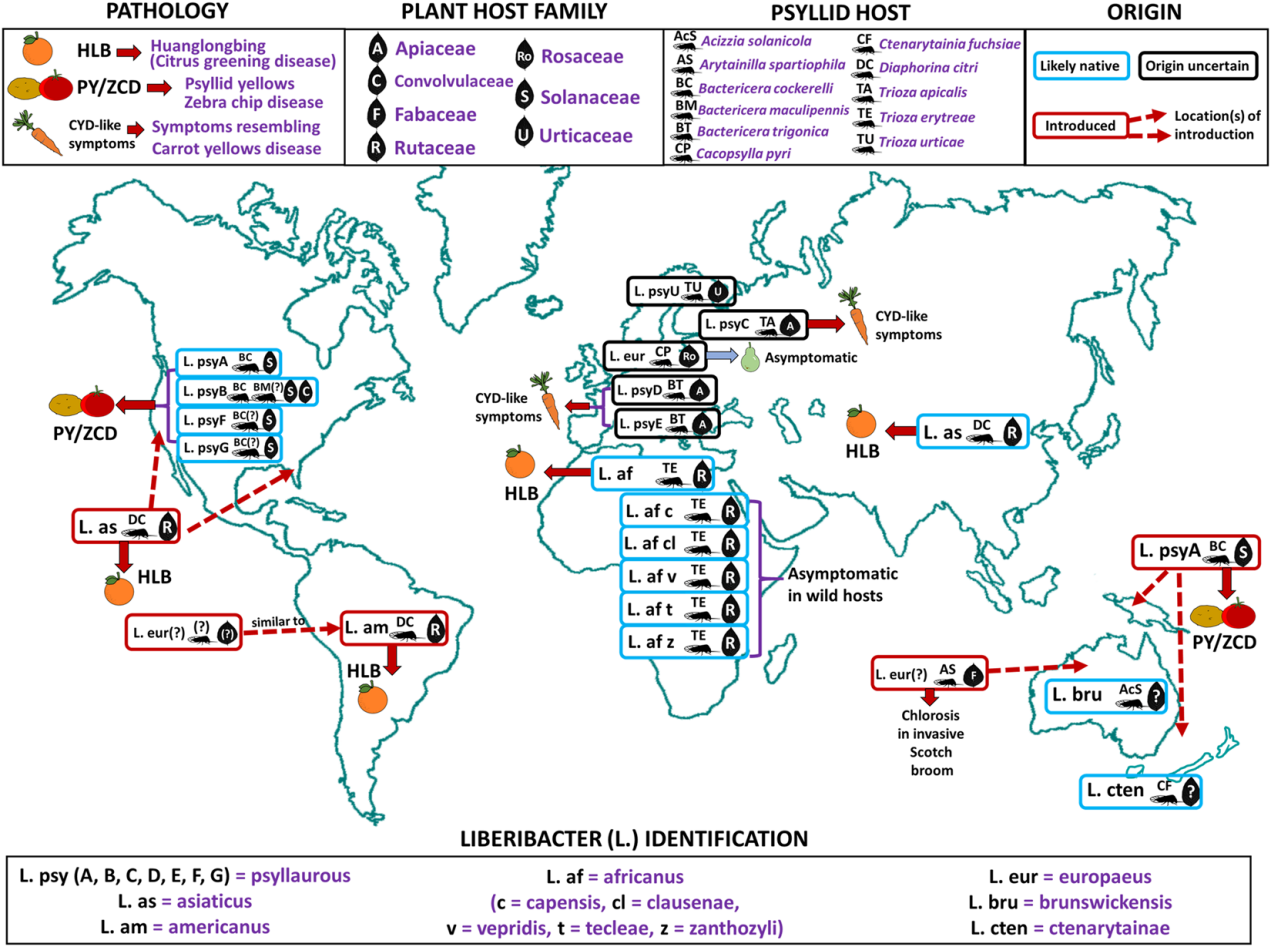
Summary of current knowledge of Candidatus Liberibacter species and haplotypes, geographic distributions, host associations (plant and psyllid), and pathology. In this study, we focus on the putative causal agent (Ca. L. psyllaurous) of psyllid yellows disease (first described in 1928^35^) and zebra chip disease (first observed in 1994^57,58^). The link between Ca. L. psyllaurous and psyllid yellows disease was first documented in 2008^17^ and shortly thereafter, was linked to zebra chip disease^18^. Information on known haplotypes of Ca. L. psyllaurous and other Ca. Liberibacter species was assembled from this study (Ca. L. psyllaurous haplotype G) and from^9^,^24^–^34^,^74^. Designed by Authors from Flaticon and Pixbay with license permission. Psyllid Icon made by Freepik from www.flaticon.com.

Among plant pathogens that have emerged as serious threats to food production in the last 100 years are those within the taxon *‘Candidatus’*(*Ca*.) Liberibacter^9^ (Fig. 1). *Ca*. Liberibacter species are largely unculturable members of the Alphaproteobacteria class in the Proteobacteria phylum^10^. Over the last decade, eight species of *Ca*. Liberibacter have been identified around the world following emergence as phloem-limited plant pathogens in mostly crop systems (cited within Fig. 1). Each known *Ca*. Liberibacter species is also associated with one or more herbivorous insect hosts (psyllids) in the superfamily Psylloidea (cited within Fig. 1). Despite the economic importance of this group, as well as the near certainty of additional emergence events, our knowledge of *Ca*. Liberibacter genetic diversity, genetic hallmarks of pathogenicity, and/or associations with insect and plant hosts comes almost exclusively from strains that produce disease in crops (cited within Fig. 1). As a result of this narrow focus, we lack critical information on *Ca*. Liberibacter species that associate with plants without being pathogenic, as well as species that may only reside and replicate within psyllid hosts. This knowledge gap can only be addressed by increasing efforts to perform diagnostic and genetic characterization studies using historical specimens and sampling in unmanaged habitats^5,11^. Such approaches are yielding a wealth of information on the diversity and epidemiology of other plant pathogen taxa across different landscape types^5,7,12–16^. However, to date there have been no efforts to discover and characterize *Ca*. Liberibacter organisms using preserved wild plant samples despite the potential for such studies to yield key information about *Ca*. Liberibacter diversity and host associations across agroecological interfaces.

The present study directly addresses this need through discovery and characterization of *Ca*. Liberibacter diversity in herbarium samples of native plants ranging from three to over 100 years old. For a target pathogen, we chose to focus on the cosmopolitan species, *Candidatus* L. psyllaurous^17^ (also known as *Ca* Liberibacter solanacearum^18^), which infects host plants belonging to the families Solanaceae^17^, Apiaceae^19^, and Urticaceae^4^. *Ca*. L. psyllaurous was first identified and named based on its association with psyllid yellows disease symptoms in potato and tomato^17,18,20^. A year following characterization, a publication named the same *Candidatus* species of bacterium ‘*Ca*. L. solanacearum’^18^. Consistent with standard ethical practices in scientific conduct, we have chosen to use the first published name ascribed to this organism^17^. The initial characterization of *Ca*. L. psyllaurous established that this pathogen is vertically transmitted in its vector, the native potato/tomato psyllid, *Bactericera cockerelli*, and subsequent work has shown that it is also harbored within and vectored by the native psyllids *Trioza apicalis*^21^, *Trioza urticae*^4^, and *Bactericera trigonica*^22,23^ in Europe. Currently, a total of 7 *Ca*. L. psyllaurous haplotypes are described throughout North and Central America, Europe, and New Zealand (Fig. 1)^4,9,24–34^.

*Ca*. L. psyllaurous is an excellent target for elucidating *Ca*. Liberibacter presence and diversity in historical specimens because it has been putatively associated with two pathological conditions in solanaceous crop hosts in North America over the last century. These are the diseases “psyllid yellows^17^” and “zebra chip^32^”. Psyllid yellows disease was first described and named in 1928 by Richards^35^, who observed characteristic yellowing symptoms in potato when in association with *B. cockerelli*. Psyllid yellows disease has been documented as “the most severe disease of potato” throughout the western USA (except for Oregon and Washington) since at least 1911, and has also been reported to infect tomato^36–50^. Following description^35^ it was hypothesized that psyllid yellows disease was either caused by a virus^51,52^ or toxins produced by the psyllid^53^. However, in early attempts to characterize this disease, induction of symptoms in new hosts was demonstrated by both grafting^38^ and tubers^50^. While PCR diagnostics were not available to confirm a causal agent, these studies suggest that direct participation of the psyllid (and any toxins deriving from it) may not be necessary for appearance of symptoms in new hosts. Evidence of a *Ca*. Liberibacter species as a possible causal agent did not come until 2008, when *Ca*. L. psyllaurous was described and named based on its association with psyllid yellows disease in potato and tomato^17,18,20^. Several subsequent studies verified transmission of psyllid yellows disease symptoms in tomato by grafting^54^, providing additional evidence in support of *Ca*. L. psyllaurous being a putative causal agent.

The more recently described zebra chip disease exhibits a very similar pathology to psyllid yellows disease^32^, including various deformities within potato tubers reported to be associated with psyllid yellows in early reports^52,55–57^. Despite this, zebra chip disease of potato was not formally documented in North America until 1994, when potato fields exhibiting characteristic tuber pathology were found in Saltillo, Mexico. The first report of this disease in the U.S.A. was in the lower Rio Grande Valley in Texas in 2000^58^. Like psyllid yellows disease, zebra chip disease is also associated with *B. cockerelli* feeding^57^ and in 2010, *B. cockerelli*-transmitted *Ca*. L. psyllaurous was found to be associated with zebra chip pathology^32^. Currently, there are two haplotypes of *Ca*. L. psyllaurous (A and B) known to occur in potato and tomato crops as well as some native host plants of the psyllid^59–62^ and the disease is present throughout the major potato production areas of Western North America.

Much of what we know about the psyllid yellows disease condition comes from historical documentation of outbreaks that occurred before the advent of diagnostic tools to detect fastidious plant pathogens. In contrast, zebra chip disease symptoms (particularly the tuber-associated pathologies) began appearing in North American potato production around the year 2000^58^ and links between these symptoms and *Ca*. L. psyllaurous have been verified using PCR in most studies performed since 2008. For example, contemporary documentation of pathologies caused by field-collected psyllids with and without *Ca*. L. psyllaurous has shown that in one potato cultivar, psyllid yellows disease and zebra chip disease share many pathological features, but additional pathologies associated with zebra chip are only evident when the psyllids feeding on the host have levels of *Ca*. L. psyllaurous detectable by standard PCR^32^. While this study does provide support for *Ca*. L. psyllaurous as a causal agent of zebra chip, it does not conclusively demonstrate a lack of association between psyllid yellows and *Ca*. L. psyllaurous because only one host and cultivar was used, making it difficult to generalize across historical accounts and contemporary reports of *Ca*. L. psyllaurous presence in other solanaceous hosts with yellows symptoms. Additionally, subsequent work using 16S ribosomal RNA pyrosequencing of DNA from field-collected psyllids (such as those used for the *Ca*. Liberibacter-free treatment in^32^) has shown that relying on PCR alone to screen psyllids for *Ca*. L. psyllaurous can lead to false negatives^63^. As a result, it is not possible to rule out involvement of a *Ca*. Liberibacter species whenever PCR is used to verify lack of infection in field-collected source insects^32^. These diagnostic challenges and an overall complicated pathology have produced an incomplete understanding of the evolutionary history of *Ca*. L. psyllaurous in North America, with knowledge gaps deriving from a lack of information about pathogen diversity and host associations prior to the introduction of molecular tools for fastidious pathogen detection.

Preservation of crop tissue in herbariums is rare, making it difficult to track pathogen evolution or associations with disease symptoms prior to the introduction of diagnostic tools. But extensive collections of native host plants of the psyllid vector, *B. cockerelli*, are available from sites in and around major potato production regions and over-wintering sites of the psyllid. Furthermore, early efforts to mitigate the threat of *B. cockerelli* have provided a wealth of natural history information about non-crop hosts exploited by this insect^64–68^. In the present study, we recovered DNA from a collection of seventy herbarium specimens consisting of three wild plant species known to serve as hosts for *B. cockerelli* (*Solanum elaeagnifolium, S. americanum*, and *S. umbelliferum*)^67,68^ and screened extracted DNA for the presence of *Ca*. L. psyllaurous. For positive samples, we determined the relationship of recovered *Ca*. L. psyllaurous sequences to other known *Ca*. L. psyllaurous haplotypes and sequence types using multilocus sequence typing (MLST) and phylogenetic approaches.

## Results

### Isolation and identification of *Ca*. Liberibacter from herbarium samples

Herbarium specimens dating back to 1910 that were collected throughout California and other southwestern states were acquired from the University of California, Riverside’s Herbarium (Supplemental Table 1; UCR 2019 (http://ucr.ucr.edu/vascularsUCR_index.php). The wild solanaceous host plants chosen for this study are frequent colonizers of disturbed natural areas in Southern California, U.S.A. (*Solanum elaeagnifolium, Solanum umbelliferum*, and *Solanum americanum*). All three of these species are documented as non-crop hosts for *B. cockerelli*^65,67,69^. *S. umbelliferum* is a drought-tolerant, summer-deciduous perennial that supports feeding by the adult *B. cockerelli*^68^. Currently it is unknown if this plant species supports the reproduction and development of psyllids, and/or harbors the *Ca. L. psyllaurous* bacterium. *S. elaeagnifolium* is a rhizomatous perennial native to the southwestern U.S.A. and has recently been identified as a suitable host for both *B. cockerelli* and *Ca. L. psyllaurous*^61,70^. *S. americanum* is an annual host suitable for *B. cockerelli* reproduction but has not yet been reported to harbor the *Ca. L. psyllaurous* bacterium^67,68^. For each plant species, all available samples were included prior to the year 1960 (corresponding with major outbreaks by *B. cockerelli* and psyllid yellows disease in potato^35,47,49,53,71^ as well as a selection of post-1960 specimens (about 2 per decade per species)) collected within key potato production regions in Southern California (Supplementary Table 1).

Based on the criteria above, 70 herbarium samples were screened with the Liberibacter primer set Las606/LSS-2 (having a 0.5 kb amplicon size within the 16S rRNA region that is specific for either *Ca*. Liberibacter africanus or *Ca*. Liberibacter psyllaurous, see methods and Supplementary Table 2). Extracts from four *Solanum umbelliferum* samples (Herbarium 51, 54, 59, 61, from the years 1995, 1999, 2011, 2016, respectively) produced positive PCR bands for this primer set whereas the remaining 66 samples and negative controls did not produce PCR bands.

Short PCR fragments (0.1–0.3 kb) are known to amplify more frequently from older, more degraded DNA samples from archived herbarium material^72^. Therefore, all extracts were also screened with the primer set Lso941/LSS-2 (having a 0.16 kb amplicon size within the 16S rRNA region that is specific for either *Ca*. Liberibacter africanus or *Ca*. Liberibacter psyllaurous, see methods). In addition to the four positive herbarium samples identified previously with the Las606/LSS-2 primer set, an additional sample (Herbarium 46 from year 1970) produced a positive PCR band using the Lso941/LSS-2 primer set. All other samples that were negative previously including the negative controls were negative for this primer set as well. To amplify a longer 16S rRNA product (1.1 kb) for BLAST and phylogenetic analyses, the primer set OA2/OI2c was used to amplify a longer fragment of the 16S rRNA region from the five positive samples (Herbarium 46, 51, 54, 59, 61). This longer fragment from the 16S rRNA region was successfully amplified in three samples (Herbarium 54, 59, 61) but not from the two oldest samples (Herbarium 46 and Herbarium 51). The longest PCR amplicons obtained from all positive samples (Herbarium 46, 51, 54, 59, 61) were Sanger sequenced and used for BLAST and phylogenetic analyses below.

Based on NCBI BLAST results, the best Blast hit that was significant (E-value = 0) for all positive amplicon sequences, from Herbarium samples (46, 51, 54, 59, 61), was the taxon *Ca*. L. psyllaurous with $\ge$99% sequence identity (Supplementary Table 3). Although all the Herbarium isolates matched *Ca*. L. psyllaurous with high sequence identity each Herbarium isolate appeared distinct from one another, because of single nucleotide polymorphisms (Supplementary Table 4). Phylogenetic analysis of the partial 16S rRNA gene supported BLAST results, and all herbarium samples clustered with *Ca*. L. psyllaurous isolates (75% branch support). Isolates 54, 59, and 51 were supported as a clade with 58% branch support (Supplementary Fig. 1). However, the 16S rRNA gene cannot fully resolve strain types^73^, consequently additional MLST analyses were performed to characterize new putative haplotypes and sequence types similar to^59^ and^4^.

### Phylogenetic analysis of *Ca*. L. psyllaurous DNA from herbarium samples

**Figure 2:**
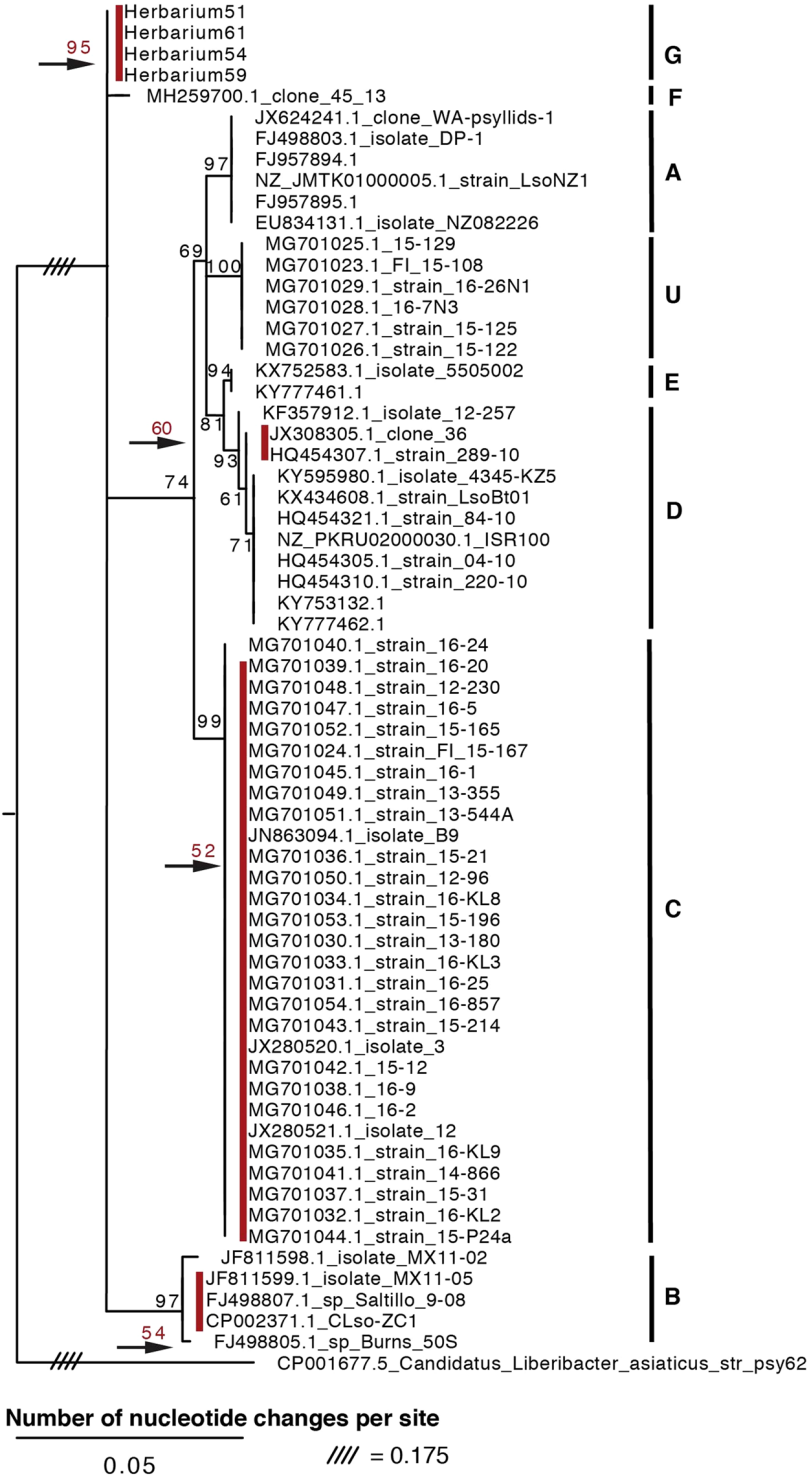
Phylogenetic relationships of *Ca*. Liberibacter psyllaurous strains and isolates based on a 605-nt alignment of the 50S ribosomal protein rplJ/rplL loci using RAxML with 100 bootstraps. The tree was rooted with the outgroup *Ca*. Liberibacter asiaticus. Only branch support at 50% or above is shown. The red bootstrap values above the black arrows, and next to the red bars, indicate a supported clade that could not be viewed given the scale of nucleotide changes. The haplotypes of strains were determined by previous studies and are indicated by the letters to the right.

Following previous approaches for determining Liberibacter haplotypes^59^ the 50S ribosomal protein rplJ/rplL loci and the 16S-23S intergenic spacer region for herbarium isolates that were positive for *Ca*. L. psyllaurous were analyzed. The 50S ribosomal protein rplJ/rplL loci were successfully amplified from extracts of herbarium samples 54, 59, 61, and 51 but not from the oldest sample (46). Surprisingly, recombination analysis of the rplJ protein (L10) revealed high levels of potential recombination in 7 regions throughout the gene for the majority of strains and within two regions for the rplL protein (L12) (Supplementary Table 5). These predicted regions of recombination are only hypothetical areas of recombination within the rplJ/rplL loci, however future studies should be cautious in using these loci for L. psyllaurous haplotyping. Nevertheless, a phylogenetic analysis using the 50S ribosomal protein rplJ/rplL loci was conducted to place results in this study in the context of previous studies^59,74^, and to ensure inclusion of isolates for which only the 50S loci were sequenced. Based on this phylogeny, the herbarium isolates formed a clade together with 95% branch support, and previously described haplotypes were found as divergent clusters supported by high bootstrap support (Fig. 2). A recently discovered haplotype, F, in the U.S.A. is most closely related to the herbarium isolates based on nucleotide identity of the 50S loci and the partial 16S rRNA sequences (Supplementary Tables 3 and 6). However, this haplotype F strain (clone 45_13) does not cluster together with the herbarium isolates based on the phylogenetic analyses of the ribosomal protein rplJ/rplL loci or the 16S rRNA loci (Supplementary Fig. 1 and Fig. 2). Collectively these results suggest that the herbarium isolates sequenced in this study belong to a different haplotype relative to previously described haplotypes, which we define here as haplotype “G”. Single nucleotide polymorphisms among the different *Ca*. L. psyllaurous isolates for the ribosomal protein rplJ/rplL loci are presented in Supplementary Table 6.

The 16S-23S intergenic spacer region (IGS) evolves rapidly and has been used in previous studies in addition to the 50S ribosomal protein rplJ/rplL loci to detect haplotypes^59^. It is important to note however that this non-coding, rapidly evolving IGS region was originally amplified with the primers Lp Frag 4-1611F and LP Frag 4-480R in *Ca*. L. psyllaurous for the detection of this new Liberibacter species in insects and plants not for the purpose of haplotyping^17^. The IGS region was successfully amplified from herbarium samples 54, 59, and 61 but not from the two oldest samples (51 and 46). Single nucleotide polymorphisms among the different *Ca*. L. psyllaurous isolates for the IGS region are presented in Supplementary Table 7. Based on the phylogenetic analysis, herbarium isolates 54, 59, and 61 clustered into a group with 97% branch support (Supplementary Fig. 2). In contrast to the 50S ribosomal protein rplJ/rplL phylogeny, the IGS phylogeny suggests very different relationships among previously characterized isolates and the herbarium isolates sequenced in this study. For example, isolates from different “haplotypes”, which were previously described, cluster together with reliable branch support (Supplementary Fig. 2). These results are consistent with those reported in Haapalainen *et al*.^4^ and suggest that either this section of the IGS region is not reliable for determining strain haplotype relationships, or, alternatively, that the 50S loci are not useful for producing a robust phylogeny. To test this further we carried out a multi-locus strain-typing (MLST) analysis.

The MLST approach previously performed by Haapalainen *et al*.^4^ was conducted to further understand how *Ca*. L. psyllaurous isolates and herbarium samples in this study are related to each other. The MLST loci (Adenylate kinase (F) gene (adk), F0F1-type ATP synthase alpha subunit (C) gene (atpA), Fructose-1,6-bisphosphate aldolase (G) gene (fbpA), Cell division GTPase (D) gene (ftsZ), Glycine/serine hydroxymethyl-transferase (E) gene (glyA), Chaperonin, HSP60 family (O) gene (groEL), and Type IIA topoisomerase, B subunit (L) gene (gyrB)) were successfully amplified from DNA extracts from the Herbarium samples 51, 54, 59, 61. Recombination was detected in adk for the fragment amplified using the adk-1F and adk-1R primers but not within the gene region using the adk- F and adk- R primers (Supplementary Table 5). Therefore, all MLST analyses were conducted using only the adk gene region amplified by adk- F and adk- R primers. All other MLST loci did not have potential recombination detected except for gyrB (Supplementary Table 5), for which recombination was detected within the first 60nt of the gyrB gene fragment. Trimming this region eliminated potential recombination from all included sequences. MLST analyses were performed using this trimmed version of the gryB gene region. Genetic variations found across all isolates for each MLST locus can be found in Supplementary Tables (8–14).

**Figure 3:**
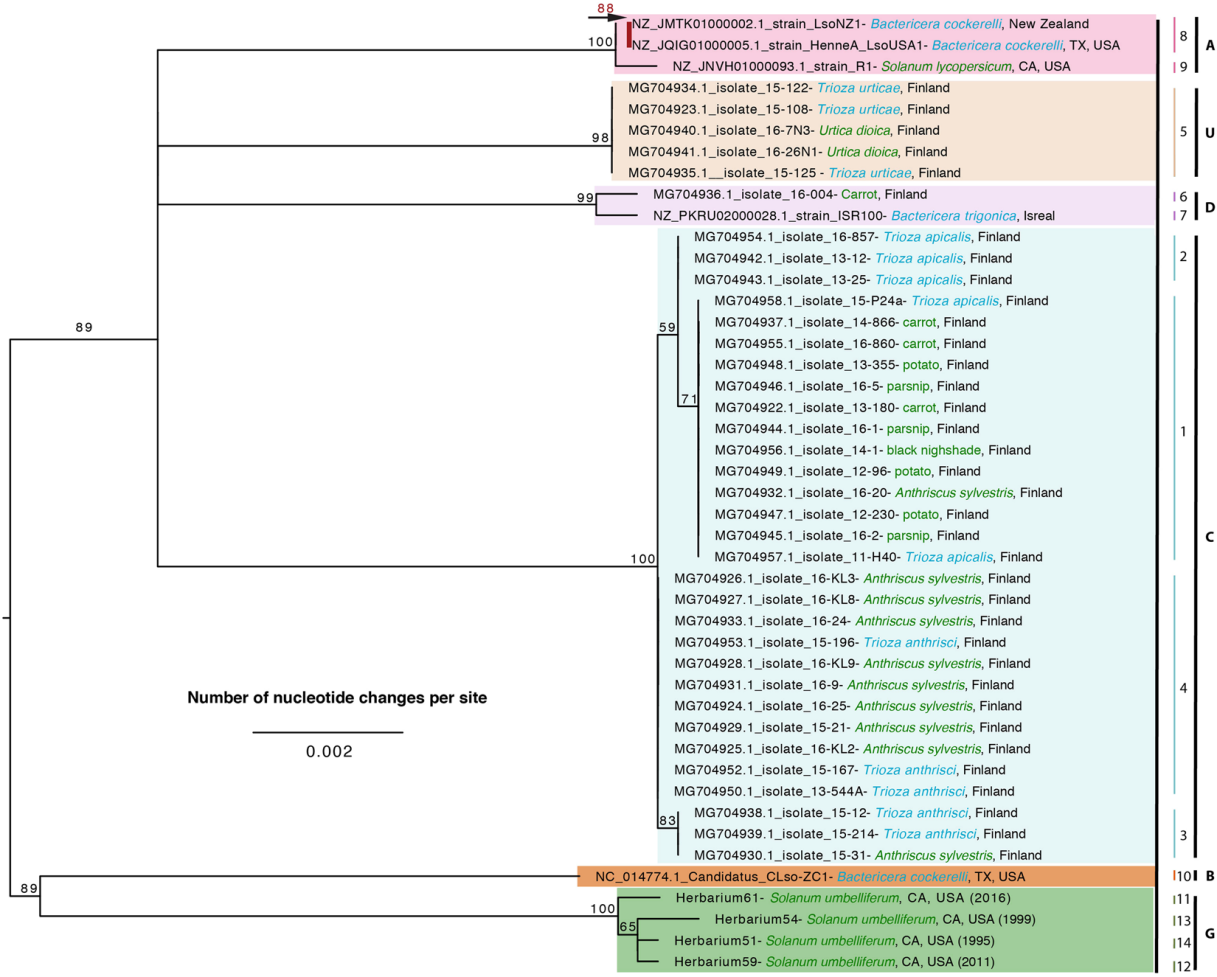
Phylogenetic relationships of *Ca*. Liberibacter psyllaurous strains and isolates based on a 3,680-nt alignment with midpoint rooting from seven concatenated, non-recombining MLST loci (adk, atpA, fbpA, ftsZ, glyA, groEL, and gryB) using RAxML with 100 bootstraps. Only branch support at 50% or above is shown. The red bootstrap value above the black arrow next to the red bar indicates a supported clade that could not be viewed given the scale of nucleotide changes. The source of each strain or isolate is indicated after the designation with color coding to indicate psyllid (blue) or plant (green) tissue source. The geographic origin of each strain/isolate is indicated after the name of the source organism. Numbers along the right side of the figure indicate the sequence type as determined by MLST strain typing in this study. The haplotypes of strains and isolates reported in previous studies^4^, as determined using a similar MLST strain typing approach, are indicated by branch colors and corresponding capital letters along the right side of the figure.

Phylogenetic analysis of the seven MLST loci revealed distinct clustering of the previously defined haplotypes (as supported by the 50S phylogeny) with branch support above 59% (Fig. 3). Based on branch length all haplotypes appear to be distantly related to one another suggesting that they have been genetically isolated for quite some time. The two haplotypes identified from North America (A and B) in potato, tomato, and the psyllid *Bactericera cockerelli* are genetically distinct from one another in addition to the newly identified haplotype in this study (G), which was isolated from non-crop host plants native to North America.

### Sequence typing of *Ca*. L. psyllaurous herbarium isolates

**Figure 4:**
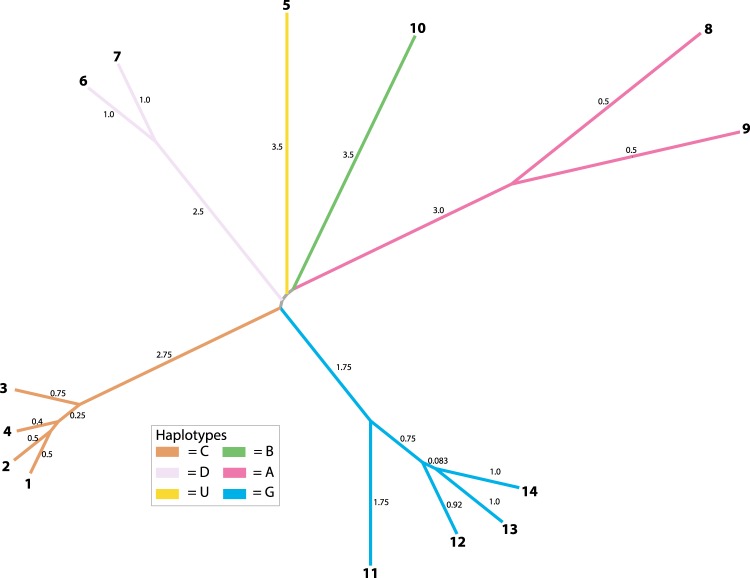
Sequence type relationships of *Ca*. Liberibacter psyllaurous strains based on allele identifiers from seven MLST loci (adk, atpA, fbpA, ftsZ, glyA, groEL, and gryB) using Neighbor-Joining Saitou-Nei Criterion. Numbers on branch tips indicate sequence type and numbers on branches indicate distance based on Hamming distance.

Sequence typing (ST) MLST analysis was conducted with the same seven loci to determine what STs most closely matched our Herbarium isolates. For each previously characterized ST, we obtained the same allele identifier numbers and used the same ST criteria for the seven loci that were developed in^4^. Based on the MLST ST criteria (see methods for details) four new STs were identified in this study (STs = 11, 12, 13, and 14, corresponding to Herbarium samples 51, 54, 59, and 61, respectively) (Supplementary Tables 1, 15). Neighbor joining analysis of these STs produced a pattern similar to our MLST phylogenetic analysis (Fig. 4). For example, STs that belong to the same haplotypes were closer to one another in distance. Moreover, the new STs (11, 12, 13, and 14) were more closely related to one another based on distance compared to the previously sequenced STs (Fig. 4). In addition, all haplotypes appear to be distantly related to one another.

## Discussion

Using herbarium specimens of native Solanaceae that were collected over the last century, we identified a new *Ca*. L. psyllaurous haplotype, “G” and four new sequence types (STs = 11, 12, 13, and 14) from preserved tissues of *Solanum umbelliferum*, a native perennial host plant of *B. cockerelli*^68^. The new haplotype G and STs were present in *S. umbelliferum* specimens originally collected from wild habitats embedded in southern California potato-growing regions and ranged from three to 49 years old. Our MLST analyses of *Ca*. L. psyllaurous haplotypes that have sequence data available for all loci (A, B, C, D, and U), and our new haplotype (G) suggests that all haplotypes are highly divergent from one another, similar to results reported by Haapalainen *et al*.^4^ (Fig. 4). Both analyses suggest that these haplotypes, which have been identified on multiple continents, have been genetically isolated from each other for quite some time (Fig. 1). This remains the case even for haplotypes identified within the same country (e.g. haplotypes A, B, and G in western U.S.A., and C and U in Finland). Based on the phylogenetic analysis of MLST loci in this study, the new haplotype G is hypothesized to share a very distant common ancestor with haplotype B, which is also found in Western North America and Central America. In contrast, haplotype A, which co-occurs with B and G in North America, appears to share a very distant common ancestor with the other haplotypes that were identified from Europe (Fig. 3).

Recently, a new haplotype, F, was discovered in diseased potato tubers from North America^33^. Based on 16S and 50S rRNA phylogenetic analyses in this study, haplotype F appears to be more closely related to haplotype G than to any other *Ca*. L. psyllaurous haplotype identified to date (Fig. 2, STs = 11, 12, 13, and 14; Supplementary Table 3). Due to its recent characterization, additional sequence data of this isolate are not publicly available at this time, so it is not possible to determine if haplotype G shares a more recent common ancestor with haplotype F compared to other L. psyllaurous haplotypes. Increasing sampling efforts to elucidate diversity within haplotype F and G would help address whether or not the pathogenic haplotype (F) that causes disease in potato^33^ has recently evolved from haplotype G, which, from this study, is only known to cause asymptomatic infections in *S. umbelliferum*.

In addition to providing a possible origin for the emergence of pathogenic haplotype F, the discovery of the G haplotype in an herbarium sample from the 1970s provides evidence that *Ca*. L. psyllaurous was present in the U.S.A. at least 30 years before zebra chip disease was reported in this country^58^. Currently, it is unclear if historic zebra chip and psyllid yellows outbreaks were caused by the same or different sequence types of *Ca*. L. psyllaurous. Zebra chip and psyllid yellows diseases may in fact be caused by the same sequence types of *Ca*. L. psyllaurous but simply manifest as different pathologies due to variation in crop cultivars, environmental conditions, crop responses to psyllid feeding, and cultivation practices. For example, the symptoms observed for psyllid yellows disease in potato vary dramatically in severity depending on the potato/tomato cultivar^66,75–77^, the number and life stages of feeding psyllids, temperature, light, soil moisture, soil alkalinity, presence/absence of extreme weather events, and whether or not the plant was co-infected with viruses and/or fungi^38,65,71,78–80^. Because samples of crop tissue from these historic outbreaks are not available, the only way to obtain information about the diversity of putative causal agents is through screening DNA from the vast repositories of preserved non-crop hosts collected in and around historic outbreak events.

**Figure 5:**
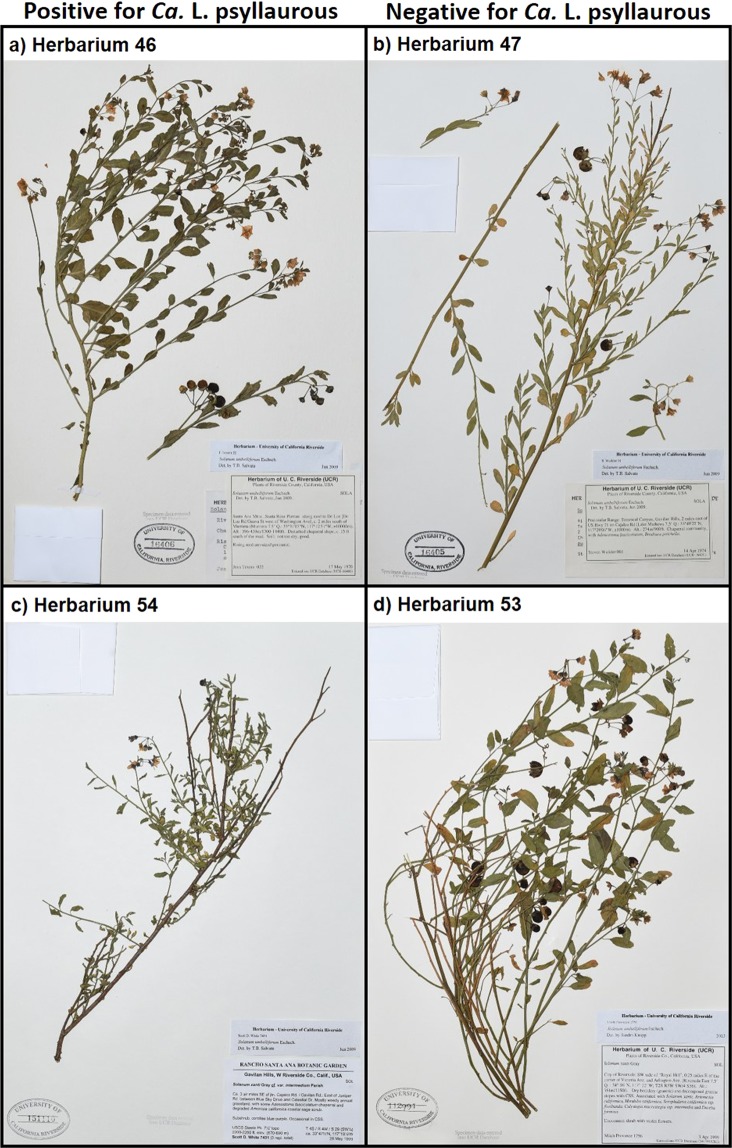
Example herbarium specimens that tested positive (left) or negative (right) for *Ca*. L. psyllaurous infection. Across all specimens that tested positive, there were no obvious signs of pathology consistent with symptoms of zebra chip disease or psyllid yellows disease, as observed in cultivated solanaceous hosts.

This study demonstrates the utility of this approach for identifying a new *Ca*. Liberibacter haplotype and strain types, as well as for developing hypotheses about the relationships among pathogenic and non-pathogenic *Ca*. Liberibacter variants. Although three known hosts of the *Ca*. L. psyllaurous vector were screened in this study, infections were detected in only one species (*S. umbelliferum*). Moreover, we did not see evidence of pathology associated with these infections (Fig. 5). *Solanum umbelliferum* has been reported as a good host for adult *B. cockerelli* feeding^68^ so it is not surprising that we detected positive infections. However, our approach of using preserved specimens does not allow us to discern why we only detected *Ca*. L. psyllaurous infections in this host and not the other two species, both of which are also good hosts for adult *B. cockerelli* feeding^68^. No information is available on suitability of *S. americanum* for *Ca*. L. psyllaurous infection, but present-day collections and laboratory experiments with another of our target species, *S. elaeagnifolium*, have confirmed that this plant is a host for both *B. cockerelli* and an unknown haplotype of *Ca*. L. psyllaurous collected in Texas (presumed to be haplotype A or B)^61,70^. Lack of detection in a host that is known to support *Ca*. L. psyllaurous infection could be attributed to poor preservation of Liberibacter DNA in *S. elaeagnifolium*, *Ca*. Liberibacter species not being present in sampled populations, or poor suitability of this species as a host for haplotype G and other *Ca*. L. psyllaurous variants. Even though infection has not been documented in contemporary *S. americanum* populations, one or more of these sources of variation could also explain the lack of positive detections in this host. It is also notable that both *S. americanum* and *S. elaeagnifolium* have primary growing periods during the spring to summer months, when alternative solanaceous crops are abundant, while *S. umbelliferum* is in leaf and bloom during the winter, when potatoes are being harvested and resources for *B. cockerelli* are limited to non-crop environments (www.calflora.org). These life history differences, and their role in determining exposure risks, could be explored through greater sampling of preserved and contemporary populations.

Regardless of the sources of variation underlying detection results, the lack of *Ca*. L. psyllaurous detection in a known host for this pathogen suggests that researchers should exercise caution when inferring pathogen incidence or abundance from preserved host material. Despite these limitations, our study demonstrates that herbarium collections can still provide important information about host breadth, pathogen diversity, and associations of pathogens with asymptomatic infections in wild plants. Consideration of host associations is becoming more important as new cryptic species and biotypes of vector insects are described alongside novel host use patterns. For instance, early natural history studies indicated that *B. cockerelli* can complete its life cycle on over 42 species of host plants, and there are also a large number of plants other than Solanaceae that can serve as temporary hosts for the psyllid^64,67,68,81^. Today, through genetic characterization of psyllid populations, we are learning that this host breadth may be due to the presence of multiple biotypes of *B. cockerelli* extending throughout North and Central America^82–84^. The host specificity of these different biotypes is not known, but likely has played a role in the sympatric evolution of several highly divergent *Ca*. L. psyllaurous haplotypes (e.g.^4^ and this study). Similarly, specific associations between *B. cockerelli* biotypes and *Ca*. L. psyllaurous sequence types have not been established, and there are few studies exploring possible alternative vectors in the North American range (but see^26^) despite novel *Ca*. L. psyllaurous-psyllid associations existing in Europe (e.g., haplotypes C and U in Fig. 1). Our study suggests that some of these unexplored avenues can be addressed through herbarium-based screening efforts that increase our knowledge of *Ca*. Liberibacter diversity. As shown here, this approach is useful for revealing new host associations and providing missing information about mechanisms driving emergence of novel pathogenic agents.

## Methods

### Sample selection

A subset of available specimens from the collection at the University of California, Riverside (UCR) herbarium were selected with assistance from the curator, Dr. Andrew Sanders (Supplementary Table 1; UCR 2019 (http://ucr.ucr.edu/vascularsUCR_index.php). The majority of these samples are from Kern and Riverside counties because these potato growing regions are likely to experience immigration of large psyllid populations from Mexico as well as over-wintering populations^85^. All the *Solanum xanti* samples collected in or before 1960 were also included because it has been classified as the same species as *Solanum umbelliferum* based on a recent phylogenetic study^86^. In total, 21 *Solanum elaeagnifolium*, 16 *Solanum americanum*, 24 *Solanum umbelliferum*, and 9 *Solanum xanti* herbarium samples were included in this study (Supplementary Table 1).

### DNA extraction

At UCR’s Herbarium collection, each specimen was mounted and housed in its own separate paper folder and was located within herbarium cabinets. No dust or other plant debris were observed on specimens that could indicate significant movement of plant material between samples. Furthermore, prior studies with herbarium specimens have established that movement of plant material among specimens, even under ideal conditions, is not sufficient to result in false positives^12^. Small pieces (~50 mm^2^ in total) of loose herbarium leaves (attached to the main plant stem but not completely glued onto the paper) were removed using sterilized plastic forceps and put into a sterile 15 ml falcon tube. To further minimize chances of contamination, new forceps were used for each sample. Three sterilized 3-mm diameter stainless steel grinding balls were put into each 15 ml tube and the dry leaf tissue was homogenized using a Geno/Grinder® (SPEX SamplePrep) by shaking at 1100 rpm for 3 min. Approximately half of the homogenized tissue was transferred to a 2 ml sterile microfuge tube using a sterilized 140-mm disposable anti-static microspatula (VWR). A different microspatula was used for each sample to prevent contamination. The grinding balls were sterilized prior to use by immersion in a 1:10 dilution of household bleach for 5 minutes, followed by a thorough rinsing with sterile deionized water (3 washes), and 100% ethanol (3 washes) before air drying at room temperature. This procedure sterilizes the grinding balls and completely destroys contaminating nucleic acids and nucleases^87^. The forceps and micro-spatulas were sterilized similarly by immersing in bleach for 60 min.

The homogenized tissue in the 2 ml microfuge tube was extracted using sorbitol extraction buffer and CTAB nuclei lysis buffer, which have previously been used to extract DNA from herbarium specimens^88^. Briefly, 150 µl extraction buffer (0.35 M sorbitol, 0.1 M Tris, 0.005 M EDTA, pH 7.5, 0.02 M sodium bisulphite) was added and tubes were vortexed. 150 µl Nuclei lysis buffer (0.2 M Tris, 0.05 M EDTA, pH 7.5, 2.0 M NaCl and 2% CTAB) was then added followed by 60 µl of 5% sarkosyl solution. All the buffers and solutions were passed through a 0.22 µm PES membrane filter (Olympus Plastics) to remove bacteria before use. Tubes were vortexed and then incubated at 65 °C for 30 min. DNA was then purified using a spin column-based protocol developed for herbarium DNA with slight modification^72^. Specifically, samples were centrifuged for 5 min at 14,000 RPM in a microcentrifuge and the supernatants were transferred to a new 1.5 ml tube. One volume of 100% ethanol was added to the extract, mixed, and incubated for 5 min on ice. The mixture was loaded to an EconoSpin® mini spin column, centrifuged at 14,000 RPM for 1 min, and the column was washed two times with 500 µl of 70% ethanol by centrifuging at maximum speed for 1 min. The column was centrifuged for another 1 min at maximum speed to eliminate any residual ethanol and then was transferred to a new 1.5 ml tube. 100 µl TE buffer was added and incubated at room temperature for 5 min followed by centrifugation at 8000 rpm for 1 min to collect the DNA extract. DNA concentration and purity were determined by a Nanodrop 2000 spectrophotometer (Thermo Scientific), and both the yield and purity of DNA from these herbarium samples indicate that DNA was successfully extracted from plant specimens regardless of age (Supplementary Table 1).

### Primer Design for amplification of 16S rRNA in *Candidatus* Liberibacter psyllaurous

16S rRNA sequences of a representative strain of *Candidatus* Liberibacter psyllaurous (EU834130), *Candidatus* Liberibacter asiaticus (DQ778016), *Candidatus* Liberibacter africanus (L22533), *Candidatus* Liberibacter americanus (AY742824), and the corresponding sequence region of a *Solanum anguivi* chloroplast genome (MH283724), were aligned together using Clustal Omega (https://www.ebi.ac.uk/Tools/msa/clustalo/). The LSS reverse primer has been shown to be specific to *Ca*. Liberibacter asiaticus, and when used together with the Las606 forward primer (universal among *Ca*. Liberibacter asiaticus, africanus, psyllaurous, and americanus), can specifically amplify a 0.5 kb Las product^89^. Therefore, we designed an LSS-2 primer (5′- ACCCAACATCTAGATAAAATC-3′) in the LSS primer region, which should be specific to *Ca*. Liberibacter africanus and psyllaurous. The primer pair Las606/LSS-2 should specifically amplify a 0.5 kb *Ca*. Liberibacter africanus or *Ca*. Liberibacter psyllaurous product. Since short fragments (0.1–0.3 kb) have been shown to be more easily amplified from herbarium samples^72^, based on the sequence alignment, we also designed a universal forward primer Lso941 (5′-GGACGATATCAGAGATGGTA-3′) downstream of Las606, which is universal among *Ca*. Liberibacter asiaticus, africanus, psyllaurous, and americanus, but should not amplify a solanum chloroplast genome. The primer pair Lso941/LSS-2 should specifically amplify a 0.16 kb *Ca*. Liberibacter africanus or *Ca*. Liberibacter psyllaurous product. The primer pair OA2/OI2c^18^ was used to amplify a 1.1 kb *Ca*. Liberibacter psyllaurous product. The primer pairs rp01F/rp01R and LpFrag4–1611F/LpFrag4-480R were used to amplify the 50S ribosomal protein rplJ/rplL gene and the 16S-23S intergenic spacer region (IGS), respectively (Supplementary Table 2). Primers for multilocus sequence typing (MLST) were used as previously described^4^. All the MLST primer sequences used in this study are listed in Supplementary Table 2.

### Polymerase Chain Reaction (PCR)

PCR reactions (20 µl reaction volume) consisted of 1 µl of template DNA, 4 µl of 5X HF buffer (ThermoFisher Scientific), 2 µl of dNTP mix (2 mM each), 1 µl of each 10 µM primer, and 0.2 µl of Phusion DNA polymerase (ThermoFisher Scientific). The PCR protocol used for all the primer pairs was: 98 °C for 5 min for initial denaturation, followed by 40 cycles of 98 °C for 10 s, 60 °C for 30 s, 72 °C for 1 min, then 72 °C for 10 min. PCR was conducted in a Bio-Rad T100™ Thermal Cycler. For Sanger sequencing of PCR products, amplicons were purified with Mag-Bind® Total Pure NGS magnetic beads (Omega Bio-tek) or Monarch® DNA Gel Extraction Kit (New England BioLabs) when necessary. 5 µl of purified PCR product (10 ng/µl) and 1 µl of 10 µM primer was submitted for Sanger sequencing. Sanger sequencing was performed by Retrogen Inc. on a 3730xl DNA Analyzer (Applied Biosystems™) and primer sequences were removed from contigs and quality trimmed prior to analysis.

### Phylogenetic analysis

Sequences for the partial 16S rRNA, 16S-23S rRNA IGS, 50S rplJ/rplL, and the seven MLST genes ((Adenylate kinase (F) gene (adk), F0F1-type ATP synthase alpha subunit (C) gene (atpA), Fructose-1,6-bisphosphate aldolase (G) gene (fbpA), Cell division GTPase (D) gene (ftsZ), Glycine/serine hydroxymethyl-transferase (E) gene (glyA), Chaperonin, HSP60 family (O) gene (groEL), and Type IIA topoisomerase, B subunit (L) gene (gyrB))) were obtained from both NCBI GenBank and from this study (Accession numbers=MN256491-MN256530 for all the 40 sequences submitted in this study). For each locus sequences were aligned using MAFFT v7.407^90^, and then were manually aligned using the graphical user Interface, Mesquite v3.51^91^ to ensure that alignments were in the right frame for protein coding loci, and/or the right strand for RNA genes. The alignments were then trimmed using trimAL v1.2^92^ with 2 parameters, gap threshold “gt” (the minimum fraction of sequences without a gap that you require to consider a column of enough quality) and minimum coverage “cons” in the trimmed alignment (that is, the trimmed alignment will retain a given percentage of the columns in the original alignment). In this case the gt and cons were considered as 0.9 and 60 respectively. Recombination Analysis was performed with Recombination Analysis Tool (RAT)^93^ on each protein separately for the 50S rplJ/rplL loci and each MLST locus separately using the auto search options. All MLST loci that did not have sites of recombination predicted were concatenated into a single alignment for phylogenetic analyses.

The phylogenies of the partial 16S, 16S-23S rRNA IGS, 50S rplJ/rplL, and the concatenated MLST genes were estimated using RAxML version 8.2.12^94^. The MPI version was executed to run the program parallelly over many connected machines on the cluster. The “GTRGAMMA” model along with option (-f a) was used to perform rapid bootstrap analysis and to find the best scoring Maximum Likelihood (ML) tree. 100 searches from the parsimony start tree were performed. The Bipartitions tree generated from RAxML was visualized using FigTree v1.4.4^95^.

### MLST sequence typing analysis

For MLST sequence typing analysis, Allele Identifiers were generated for each of the MLST loci that were predicted to not have recombination using RAT^93^. If nucleotide sequences of the same MLST locus (gene) were identical they were assigned the same Allele Identifier. If there was variation of at least one nucleotide in a sequence it was assigned to a different Allele Identifier for the locus. To determine genetic variation between sequences for each locus a custom Python script was written to find the variations, their count and column number of the variations in each gene alignment. Each unique Allele Identifier profile for all MLST genes for each strain was then assigned a different Sequence Type (ST) number. MLST sequence typing followed a similar assignment as in^4^. The Allele Identifier profile and STs of strains were analyzed in PHYLOViZ 2.0 using the Neighbor-Joining algorithm with Hamming Distance to measure genetic distance, and the Saitou-Nei Criterion for tree branch-length minimization^96^.

## Supplementary information

Supplementary Figures 1 and 2

Dataset 1–15

## Data Availability

Sequence data from our study were deposited into NCBI Genbank with accession numbers MN256491-MN256530.
