# Author Correction: New *Ca*. Liberibacter psyllaurous haplotype resurrected from a 49-year-old specimen of *Solanum umbelliferum*: a native host of the psyllid vector

**DOI:** 10.1038/s41598-019-53785-z

**Published:** 2019-11-22

**Authors:** Kerry Elizabeth Mauck, Penglin Sun, Venkata RamaSravani Meduri, Allison K. Hansen

**Affiliations:** Department of Entomology, University of California, Riverside, 900 University Ave, Riverside, CA USA

Correction to: *Scientific Reports* 10.1038/s41598-019-45975-6, published online 02 July 2019

In the original version of this Article, the names of the authors Kerry Elizabeth Mauck, Penglin Sun, Venkata RamaSravani Meduri and Allison K. Hansen were incorrectly given as K.E. Mauck, P. Sun, V. Meduri and A. K. Hansen.

Additionally, this Article contained an error in the Methods section under the subheading ‘Phylogenetic analysis’.

“(Accession numbers = biosample SUB5094043 with SAMN10841479 for all sequences submitted for Herbarium_51, SAMN10841480 for all sequences submitted for Herbarium_54, SAMN10841481 for all sequences submitted for Herbarium_59, SAMN10841482 for all sequences submitted for Herbarium_61, and SAMN10841483 for all sequences submitted for Herbarium_46)”

now reads:

“(Accession numbers = MN256491-MN256530 for all the 40 sequences submitted in this study)”

Lastly, this Article contained errors in the Data Availability Statement.

“Data from our study were deposited into NCBI Genbank under biosample SUB5094043 with the following Accession numbers = SAMN10841479, SAMN10841480, SAMN10841481, SAMN10841482, and SAMN10841483.”

now reads:

“Sequence data from our study were deposited into NCBI Genbank with accession numbers MN256491-MN256530.”

These errors have now been corrected in the PDF and HTML versions of the Article.

