# Supplementary material for: New Ca. Liberibacter psyllaurous haplotype resurrected from a 49-year-old specimen of Solanum umbelliferum: a native host of the psyllid vector
Source: Sci Rep. 2019 Jul 2;9:9530. doi: 10.1038/s41598-019-45975-6 (PMC6606623; doi:10.1038/s41598-019-45975-6)
Supplement: Supplementary file 1 — Supplementary Figures 1 and 2 [file 41598_2019_45975_MOESM1_ESM.docx]

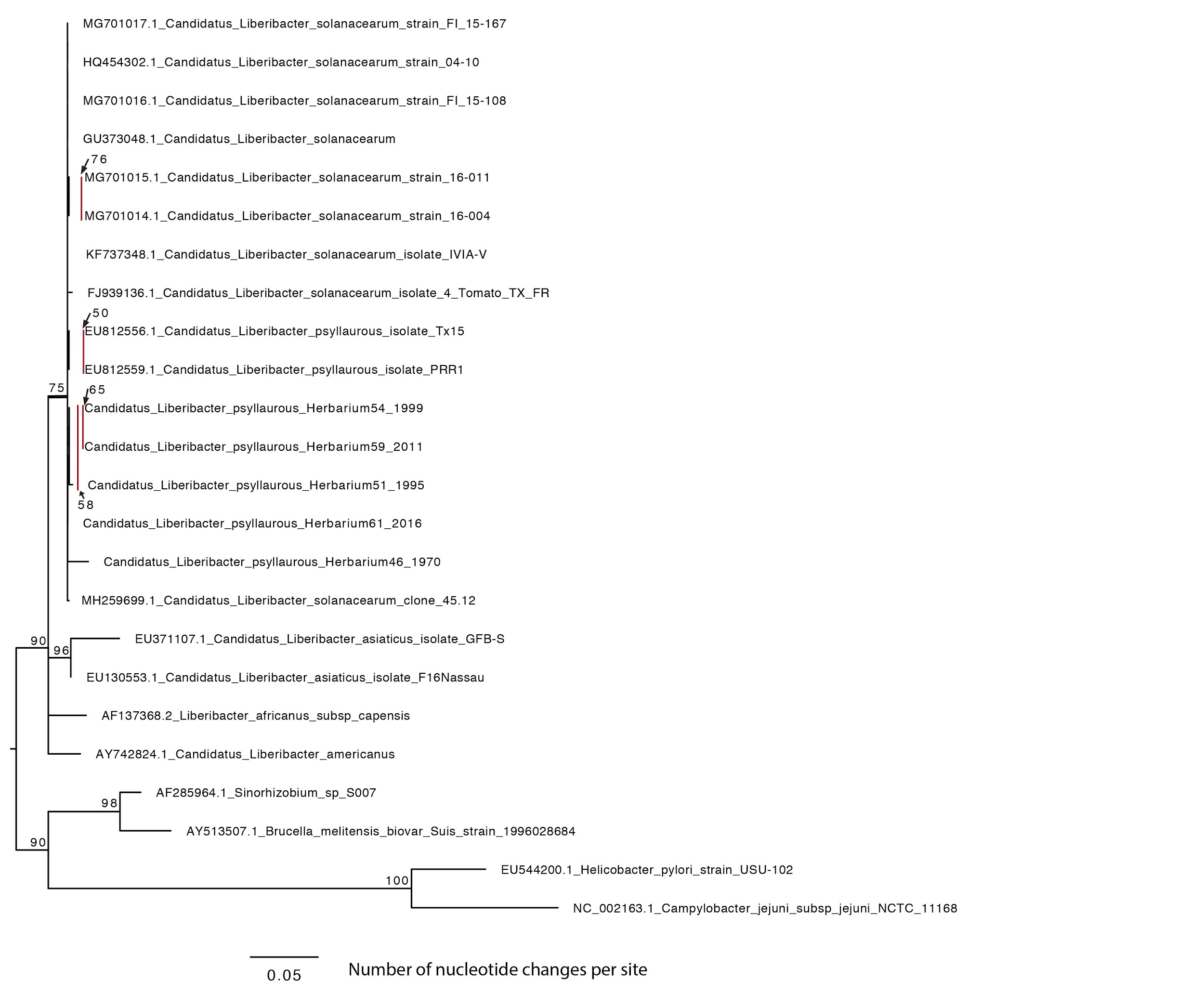


**Supplementary Figure 1.** Phylogenetic relationships of *Ca.* Liberibacter psyllaurous isolates based on a 912-nt alignment of the partial 16S ribosomal RNA loci using RAxML with 100 bootstraps. The tree was rooted with the outgroups AF285964.1, AY513507.1, EU544200.1, and NC002163.1. The bootstrap value associated with the black arrow next to the red bar indicates a supported clade that could not be viewed given the scale of nucleotide changes. Only branch support at 50% or above is shown.

**
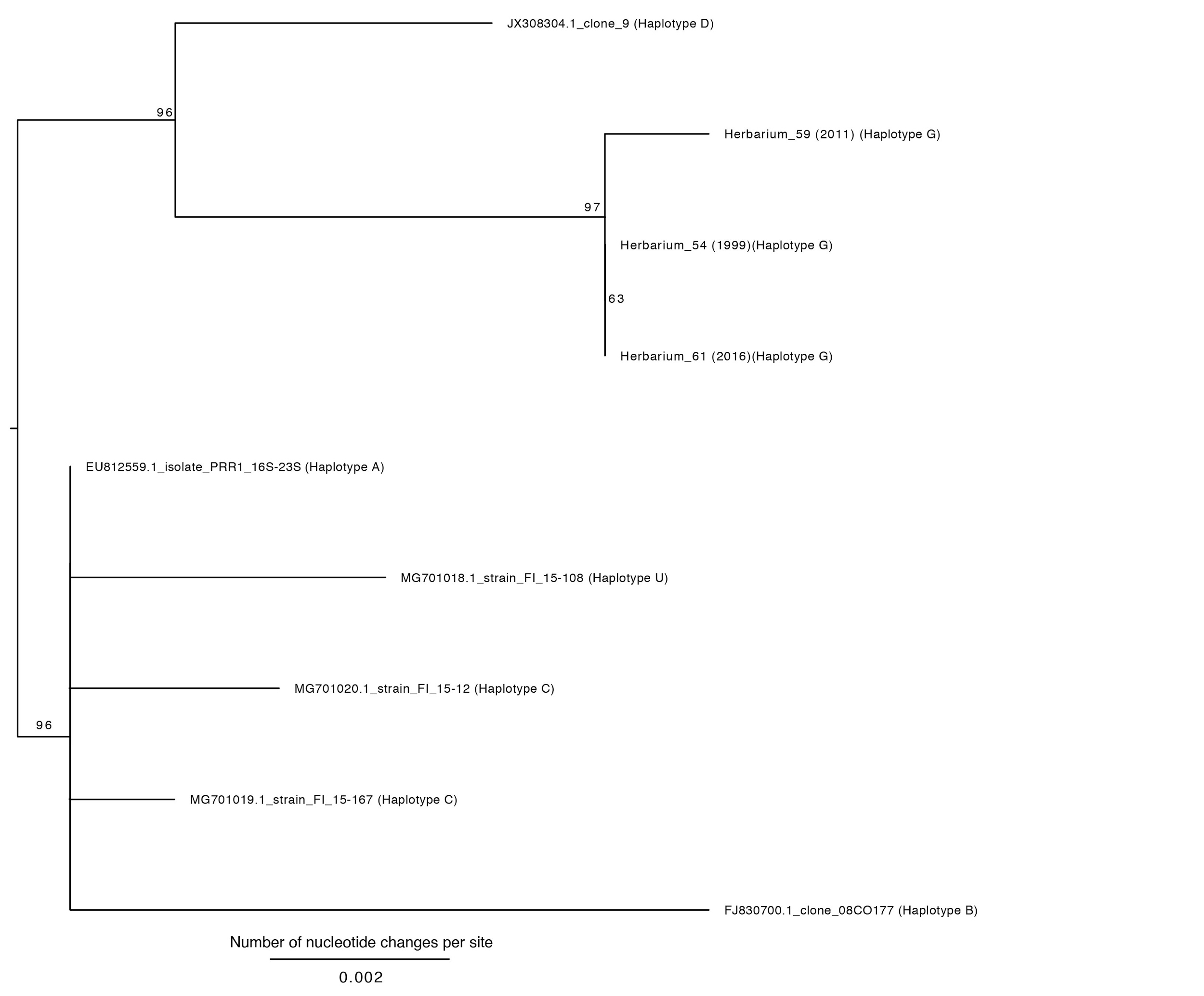
**

**Supplementary Figure 2.** Phylogenetic relationships of *Ca.* Liberibacter psyllaurous isolates based on a 864-nt alignment with midpoint rooting from the 16S-23S intergenic spacer region (IGS) using RAxML with 100 bootstraps. Only branch support at 50% or above is shown.
